# Evaluation of Sufu Fermented Using *Mucor racemosus M2*: Biochemical, Textural, Structural and Microbiological Properties

**DOI:** 10.3390/foods12081706

**Published:** 2023-04-19

**Authors:** Yuan Xie, Ziyu Guan, Shitong Zhang, Jie Zhang, Zhihui Yang, Joe M. Regenstein, Peng Zhou

**Affiliations:** 1 State Key Laboratory of Food Science and Technology, Jiangnan University, Wuxi 214122, China; 2 School of Food Science and Technology, Jiangnan University, Wuxi 214122, China; 3 Department of Food Science, Cornell University, Ithaca, NY 14853-7201, USA

**Keywords:** sufu, *Mucor racemosa M2*, fermented soybean, soybean, soyabean

## Abstract

The quality and safety of sufu fermented using *Mucor racemosa M2* was studied and compared with naturally fermented sufu. After 90 days post-fermentation, both naturally fermented and inoculated fermented sufu reached the maturity standard of sufu, and the degree of protein hydrolysis of natural sufu (WP/TP: 34% ± 1%; AAN/TN: 33% ± 1%) was slightly higher than that of the inoculated sufu (WP/TP: 28.2% ± 0.4%; AAN/TN: 27% ± 1%). The hardness and adhesiveness of inoculated sufu (Hadness: 1063 g ± 211 g; Adhesiveness: −80 g ± 47 g) were significantly greater than those of natural sufu (Hadness: 790 g ± 57 g; Adhesiveness: −23 g ± 28 g), and the internal structure of natural sufu was denser and more uniform than that of inoculated sufu. A total of 50 aroma compounds were detected in natural and inoculated sufu. The total number of bacterial colonies in naturally fermented sufu was significantly higher than that in inoculated sufu, and the pathogenic bacteria in both types of fermented sufu were lower than the limit of pathogenic bacteria required in fermented soybean products. The content of biogenic amines in sufu was determined by high performance liquid chromatography (HPLC), and the results showed that the content of biogenic amines (*Putrescine*, *Cadaverine*, *Histamine*, *Tyramine*, etc.) in naturally fermented sufu was significantly higher than that in inoculated fermented sufu. Especially the histamine content, after 90 days of fermentation, was found to be 64.95 ± 4.55 for inoculated fertilization and 44.24 ± 0.71 for natural fertilization. Overall, the quality of inoculated sufu was somewhat better than that of natural sufu, and the *M2* strain can be used to ferment sufu.

## 1. Introduction

**Figure 1:**
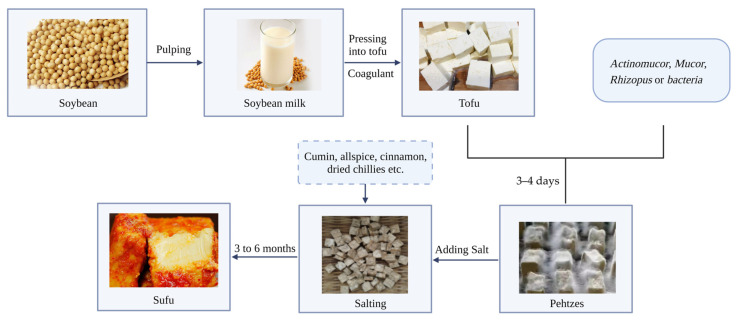
A diagram of sufu fermentation.

Sufu, also known as fermented soybean curd, moldy soybean curd or milk soybean curd, is a traditional Chinese fermented soybean product. This product is enjoyed by the majority of consumers due to its special texture, valuable nutrition and delicious flavor [1,2]. According to the fermentation strains, sufu can be divided into two types: inoculated fermentation sufu (inoculated with *Actinomucor*, *Mucor*, *Rhizopus* or *bacteria*) and natural fermentation sufu [3]. The basic processing procedure for sufu has remained almost unchanged for hundreds of years [4,5,6]. Soybean is used as the main raw material and undergoes complex physical, chemical and biochemical processes. Preparation mainly includes two steps (Figure 1): (1) preparation of tofu: soybeans are soaked and expanded and then ground, bean dregs are removed to obtain soybean milk and coagulant is added to the cooked soybean milk; (2) preparation of sufu: the mold or bacteria are inoculated on the tofu, and after culturing for 3–4 days, salting is carried out, and then auxiliary ingredients (Cumin, allspice, cinnamon, dried chilies, salt, etc.) are added and fermented for 3 to 6 months.

Fermentation of sufu involves the synergistic action of microorganisms and enzymes, which is a complex biochemical process [7]. Among these, the fermentation strain has an important role in the quality of sufu. *Mucor* are widely used in the production of sufu, accounting for >90% of all Chinese sufu, due to its well-developed mycelium [3,8]. The *Mucor* can form a uniform layer of mycelium film on the surface of the tofu, wrapping the tofu pieces, thus giving the sufu a unique texture while keeping the intact shape of the tofu. In addition, *Mucor* secretes multiple enzymes with high activity. The secreted proteases and peptidases can degrade the protein to produce peptones, polypeptides and amino acids which enhance the nutrition of sufu and contribute to its flavor [8]. *Mucor* proteases have strong peptidase activity and can hydrolyze bitter peptides into free amino acids, thereby removing or reducing the bitterness of fermented bean curd [9]. *Mucor* can also secrete a catechol oxidase, which converts the colorless flavonoids and isoflavones in sufu into yellow hydroxyl compounds, and the attractive golden yellow color stimulates the appetite [10].

Sufu is a popular food in China. Yunnan sufu is fermented using naturally available bacteria. The open production environment and uncontrol way which may lead to poor safety and quality of the products, some bacteria may bring undesirable metabolites, such as biogenic amines (BAs) [6] In this study, *Mucor racemosus* M2, a strain isolated from the natural fermentation, was used as the fermentation strain, and the sufu was analyzed and compared with the naturally fermented sufu. The purpose was to develop a high-quality and stable sufu and provide a theoretical basis for the actual production of sufu in Yunnan.

## 2. Materials and Methods

### 2.1. Materials

Soybeans were purchased from Jinyuan Wholesale Co., Ltd. (Huaibei, China). Red pepper, ground zanthoxylum and salt were from Yunnan Dianxue Grain and Oil Co., Ltd. (Yuxi, China). *Mucor racemosus* MD-102 (CGMCC No. 15264) was isolated from sufu produced in Mouding, Yunnan Province, and was identified using Polymerase Chain Reaction as described in a previous report [11]. Chemicals used were at least of reagent grade.

The sufu was prepared as described by Wei, Yang, Regenstein, Liu and Liu [12]. After soaking, soybeans were refined, boiled, pulped, pressed into tofu and then cut into small pieces (3.5 × 3.5 × 1.5 cm). For natural fermentation, these pieces of tofu were put on straw dry stalks of wheat for natural fermentation for 3 days to form pre-fermented pehtzes. For the inoculated fermentation, these pieces of tofu were uniformly sprayed of suspension via sprinkler (10^5^ spores/mL, ~0.5 mL/piece) for fermentation for 3 days. The salted pehtzes were obtained by drying and salting, mixing the pehtzes with red pepper, zanthoxylum and salt at a ratio of 100:8:0.5:10 (*w*/*w*/*w*). A solution of 4% salt and 3% ethanol prepared from 95% alcohol was added to the pehtzes, and the bottles were sealed and ripened for 180 days at room temperature (25 ± 2 °C). Samples were collected and the separated samples were analyzed at 0, 90 and 180 days of ripening and identified as R-0 d, R-90 d, R-180 d.

### 2.2. Methods

#### 2.2.1. Determination of the Main Chemical Composition

The main chemical indicators in naturally fermented and inoculated fermented sufu were determined. These indicators include moisture content, salt content, total acid and amino acid nitrogen content, protein content and water-soluble protein content. The moisture content in sufu was determined by the “direct drying method” following the Chinese National Standard (2016) (GB 5009.3-2016). The salt content of sufu was tested according to the “Faulhard method (indirect precipitation titration method)” specified in the Chinese National Standard (2016) (GB 5009.44-2016). The total acid and amino acid nitrogen content in sufu is determined in accordance with the “acidity meter method” specified in the Chinese National Standard (2016) (GB/T 5009.235-2016). Protein content in sufu is measured by the “Kjeldahl method” according to the Chinese National Standard (2016) (GB 5009.5-2016), and the total nitrogen content is expressed as protein content/5.71. The water-soluble protein content in sufu is determined in accordance with the recommended standard (2016)(NY/T 1205-2006) of the Ministry of Agriculture of China.

#### 2.2.2. Degree of Protein Hydrolysis

The total contents of nitrogen in sufu were determined using the method of Kjeldahl [13] and crude protein determined using a factor of 5.71. Ultrapure water (Healforce Biomedical Technology Co., Ltd., Shanghai, China, ≥18 MΩ, 100 mL) was added to 5 g sufu ground with a mortar and pestle and then sonicated at 280 W, 20 kHz, for 40 min in a water bath at room temperature. After filtration using filter paper, 20 mL of filtrate was mixed with 50 mL of ultrapure water. NaOH solution (0.02 mol/L) was used to titrate the mixture until pH 8.2, and the volume of NaOH solution was used for the calculation of the total acid content. After adding 10 mL of formaldehyde solution (38%), the same NaOH solution was used to titrate until pH 9.2, and the additional volume of NaOH solution was used for the calculation of amino acid nitrogen content. Ground sufu (5.0 g) was diluted to 100 mL with ultrapure water and then filtered at room temperature after sonication for 40 min. Then, 20 mL of filtrate was used for Kjeldahl digestion, distillation, and titration with 0.03 mol/L HCl. The volume of HCl was used for the calculation of the water-soluble crude protein content using the same Kjeldahl factor. An estimate of the degree of protein hydrolysis in sufu used the percentage of water-soluble to total crude protein (WP/TP) as well as amino acid nitrogen to total nitrogen (AAN/TN).

#### 2.2.3. Free Amino Acids

Trichloroacetic acid (TCA, 5%) was added to 1 g of sufu with a final volume of 25 mL and left for 4 h after 20 min of sonication. Then, the sample was filtered, and the filtrate was centrifuged at 10,000× *g* (Heraeus Multifuge X1R, Thermo Fisher Scientific Inc., Langenselbold, Germany), 20 °C, for 30 min. The supernatant was collected and filtered using a 0.22 µm syringe filter (Waters Co. Ltd., Shanghai, China) for HPLC (Agilent 1100, Agilent Technologies Co. Ltd., Santa Clara, CA, USA) analysis. Samples were injected into an Agilent Hypersil ODS column (125 × 4.6 mm × 5 µm) at 40 ℃ and detected at 338 nm (G1314A VWD, Agilent Technologies, Inc., Santa Clara, CA, USA). The mobile phase A was 20 mM sodium acetate (pH = 7.2) containing 0.5% tetrahydrofuran and mobile phase B was 20 mM sodium acetate:methanol:acetonitrile at the ratio of 1:2:2 and at a flow rate of 1.0 mL/min. A linear elution program from 8–50% mobile phase B (0–17 min), 100% mobile phase B (17–20 min) and 0% mobile phase B (20–24 min) was used. Standards (Sigma-Aldrich, St. Louis, MO, USA) were analyzed using the same conditions as with the samples and the concentration of free amino acids was determined using a standard curve.

The taste activity value (*TAV*) [14] was used to evaluate the contribution of each Free amino acids (FAA) to the taste of food. The equation of the *TAV* was:

(1) $$TAV_{i}=\frac{C_{i}}{\mathrm{TV}},$$

where *C_i_* was the concentration of each FFA and TV was the threshold of the taste substance [15]. When the value of *TAV* is >1, it means that the substance contributes to the taste of the food and, the higher the *TAV* value, the greater the contribution of the taste substance to the overall taste. On the other hand, the substance does not contribute to the overall taste of the food when the value of *TAV* is <1.

#### 2.2.4. Texture and Microstructure

Texture analysis was conducted according to the method of Czerner [16]. The texture (hardness, adhesiveness and springiness) of a pehtzes was measured with a P50 probe using a texture analyzer (TA-XT plus, Stable Micro System, Ltd., Godalming, UK). The crosshead speed before, during and after the test was 1 mm/s with a compression ratio of 30% and trigger force of 5 g.

Scanning electron microscope (SEM, FEI Quanta 200, FEI Co., Hillsboro, OR, USA) was used to observe the microstructure of sufu as described by Jiang [17]. Samples were cut using a knife into small pieces (3 × 3 × 5 mm) and placed in a 2.5% glutaraldehyde solution for 4 h at 4 °C. Then, the samples were washed 3 times using 0.2 mol/L sodium phosphate buffer (pH = 7.4), dehydrated using a gradient concentration of ethanol (30, 50, 70, 90, and 100%) (Sangon Biotech Co., Ltd., Shanghai, China) and immersed in chloroform for 2 h to remove fat and then isoamyl acetate twice for 15 min. After that, the samples were dried using a freezer drier (Advantage EL-85, SP Scientific, Inc., Gardiner, NY, USA) and coated with gold using ion sputtering (SCD 050, BAL-TEC AG, Balzers, Liechtenstein) and observed at 15 kV with 500 times magnification.

#### 2.2.5. Aroma Compounds

Aroma compounds in sufu were extracted and analyzed using headspace solid-phase microextraction gas chromatography–mass spectrometry (HS-SPME-GC/MS: HS-SPME, Thermo TSQ 8000, Thermo Fisher Scientific (China) Co., Ltd., China; GC/MS, Thermo Trace 1300, Thermo Fisher Scientific (China) Co., Ltd., China) as described by Feng [18]. For sample preparation, 5 g of ground sufu was thoroughly mixed with 1.0 g of NaCl and 5.0 g of ultrapure water. A total of 5 g of the mixture was added into a 20 mL headspace flask (Hamai Instrument Technology Co., Ltd., Ningbo, Zhejiang, China), with 20 µL of ethyl 2-phenylacetate methanol solution as the internal standard. The flask was put into a water bath at 60 °C for 10 min with shaking and preheating, followed by headspace extraction using a CAR/PDMS fiber (75 µm, Supelco Inc., Bellefonte, PA, USA) for 20 min.

Gas chromatography was conducted using a DB-WAX column (30.0 × 0.32 m × 0.25 µm), and the carrier gas was helium at a flow speed of 1.5 mL/min. The initial temperature of the oven was 40 °C. It was maintained for 2 min and then increased to 185 °C at 10 °C/min and held for 1 min, then increased to 240 °C at 10 °C/min and held for 8 min. The electron impact (El) was used with an ionization voltage of 70 eV, and the scan range was from 33 to 440 *m*/*z*, with a scan rate of 0.2 s/scan for 30 min. The aroma compounds obtained were compared with the NIST (Version 2.2) database and the compounds with similarity >80% were retained for tentative qualitative determinations and an estimation of the amount of each compound.

#### 2.2.6. Microbiology Analysis

Random samples were chosen and serially diluted using 0.85% NaCl until the proper dilution for enumeration. The total bacterial and total fungal count—*Salmonella*, *Staphylococcus aureus* and *Escherichia coli*—were measured, respectively, using Man Rogosa Sharpe, potato dextrose agar, xylose lysine desoxycholate agar, Baird Parker and violet red bile-4-methylumbelliferyl β-D-glucuronide agar.

#### 2.2.7. Determination of Biogenic Amines

A sample of 5 g was added with TCA (5%), homogenized for 2 min and sonicated for 30 min. After centrifugation at 10,000 r/min for 10 min, the supernatant was transferred to a 25 mL brown volumetric flask, and the precipitate was added with 5% TCA and the previous processing was repeated. The supernatant was combined and diluted to 25 mL with 5% TCA. The supernatant was added with n-hexane at a ratio of 1:1 and vortexed for 5 min and the supernatant was discarded after standing still to remove fat. The extract of 1 mL was added to 200 µL of 2 mol/L NaOH solution, 300 µL of saturated NaHCO_3_ solution and 2 mL of 10 mg/mL of acetone solution of dansyl chloride. It was vortexed, and the reaction was carried out for 45 min at 45 °C in a water bath protected from light and then terminated by adding 100 µL of ammonia. The mixture was fixed to 5 mL with the addition of acetonitrile and centrifuged at 5000 r/min for 5 min. The supernatant was loaded into a liquid phase bottle after passing through a 0.22 µm aqueous membrane for HPLC (Agilent 1100, Agilent Technologies Co. Ltd., Santa Clara, CA, USA) analysis.

Chromatographic conditions: The sample was analyzed using TSK gel ODS-80TM (4.6 × 250 mm, 5 µm) column with ultrapure water as mobile phase A and acetonitrile as mobile phase B. The column temperature was 30 °C. A gradient elution was used, and the elution procedure is shown in Table 1, with the flow rate of 1 mL/min, UV detection at 254 nm and a sample volume of 20 µL. The standard solution was prepared using 50 mg each of tryptamine, β-phenylethylamine, putrescine, cadaverine, histamine, tyramine, spermidine and spermine and dissolved by 0.1 mol/L HCl to make a mixed standard working solution of 80, 50, 20, 10, 5, 1, 0.5 and 0.1 µg/mL, respectively.

#### 2.2.8. Statistical Analysis

For each trial, quality analyses were triplicated. The Statistical Program for the Social Sciences, SPSS 23 (SPSS, IBM Corp., Chicago, IL, USA) was used for analysis of variance. Significance analysis of the experimental data was performed using Duncan’s multiple range test, with *p* < 0.05 being significant. Principal component analysis (PCA) of aroma compounds was conducted using XLSTAT 2016 processing (Version 2018.1, Addinsoft, Paris, France). All data are shown as mean ± standard deviation.

## 3. Results and Discussion

### 3.1. Major Chemical Indicators

**Figure 2:**
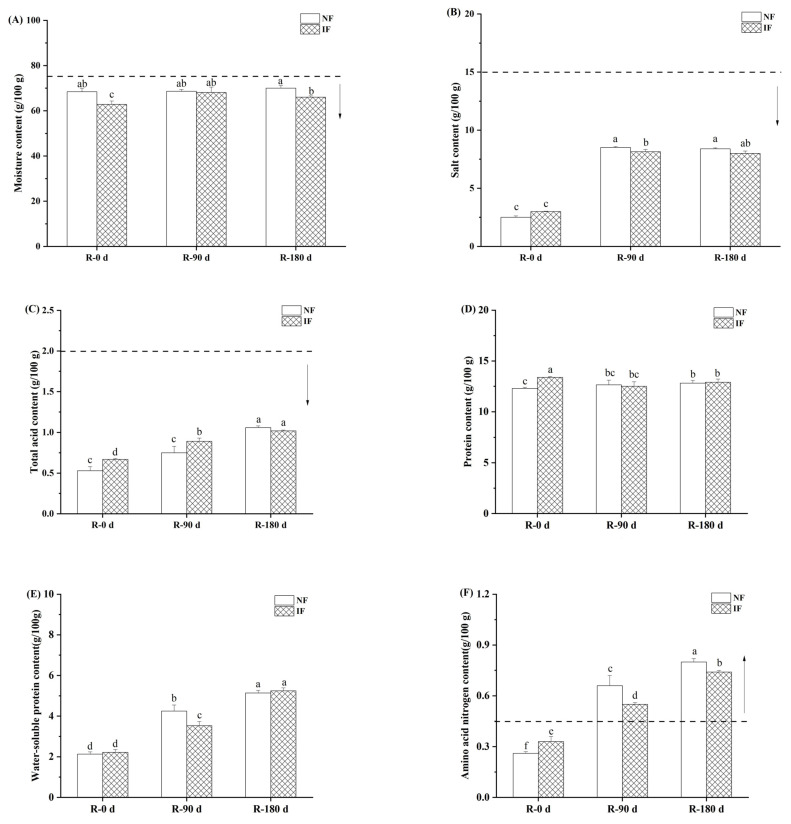
Contents of moisture (**A**), salt (**B**), total acid (**C**), protein (**D**), water-soluble protein (**E**) and amino acid nitrogen (**F**) in natural and inoculated fermented sufu. Note: Different lower-case letters indicated significant differences at *p* < 0.05. NF: natural fermentation; IF: inoculated fermentation. The dotted line is the standard requirement for each indicator of mature vegetarian curd; the arrow facing up indicates that mature vegetarian curd needs to be greater than the value; the arrow facing down indicates that mature vegetarian curd should be less than the value.

The contents of the main chemical components in natural and inoculated sufu are shown in Figure 2. The increase of moisture content in fermented food will loosen the protein network structure and weaken the bonding between protein molecules, resulting in a softer texture of the product [19]. As shown in Figure 2A, the moisture content in both natural and inoculated sufu was stabilized at 60–70% during post-fermentation, which was in accordance with the Chinese standard for locally marked products of sufu (2015) (DB53/T 713-2015) (moisture content ≤75 g/100 g), indicating that the fermentation strains had little effect on the moisture content in sufu (*p* < 0.05), which was consistent with Cui [20].

The main role of salt in the fermentation process of curd is to impart salty taste to the curd, inhibit enzyme activity, inhibit the growth of microorganisms, especially miscellaneous bacteria and pathogenic bacteria, and prevent quality problems caused by excessive fermentation of curd [1]. The salt content in sufu is shown in Figure 2B, where the salt content of natural and inoculated sufu was 2.5 g/100 g and 3.0 g/100 g, respectively, at 0 day of post-fermentation, and increased to 8.5 g/100 g and 8.2 g/100 g, respectively, at 90 days of post-fermentation. The main reason for the significant increase in the salt content of sufu is that the salt on the surface of the salt billet and in the soup penetrated into the inside of sufu during the post-fermentation process. The salt content at 180 days of post-fermentation showed less change compared with that at 90 days, indicating that the osmotic effect of salt content inside and outside the sufu billet had basically stabilized at 90 days of post-fermentation.

The total acid in curd mainly comes from amino acids, fatty acids and organic acids generated from protein, fat and carbohydrates under the action of enzymes, which is one of the important indicators of the quality and fermentation degree of curd. As shown in Figure 2C, the total acid content in sufu increased with increasing fermentation time, which is consistent with the trend of total acid content in curd fermented by *Rhizopus oligosporus* and *Rhizopus wagnerii* [20], while the difference between total acid content in sufu fermented naturally and by inoculation was not significant (*p* ≥ 0.05).

Proteins in curd are degraded into small molecules of peptides, free amino acids and ammonia through fermentation, which play an important role in the texture and flavor formation of curd [21,22], while the water-soluble protein content is closely related to the degree of protein hydrolysis. As shown in Figure 2D,E, the protein content in both natural and inoculated sufu was stable at about 13 g/100 g during post-fermentation, while the water-soluble protein content increased significantly (*p* < 0.05) with the extension of fermentation time. At 0 days of post-fermentation, the water-soluble protein content in naturally fermented and inoculated fermented sufu was not significantly different (*p* ≥ 0.05) and was around 2.1 g/100 g. At 90 days of post-fermentation, it increased to 4.25 g/100 g and 3.53 g/100 g, respectively, reaching the standard for locally marked products of sufu (Chinese standard for locally marked products of sufu marked products of sufu (2015) (DB53/T 713-2015)) (water-soluble protein content ≥ 3.5 g/100 g). The increase of water-soluble protein content was mainly due to the proteins in sufu being hydrolyzed into water-soluble small-molecule peptides and amino acids under the action of protease. After 180 days fermentation, due to the consumption of nutrients by microorganisms and the accumulation of metabolites, the increase rate of water-soluble protein content in sufu became slower, and the contents were 5.14 g/100 g and 5.25 g/100 g, respectively.

Amino acid nitrogen refers to the nitrogen element in the form of amino acid, which is the end product of protein hydrolysis. Therefore, the determination of amino acid nitrogen can further illustrate the hydrolysis of protein, and the amino acid nitrogen content (≥0.45 g/100 g) is often taken as the main indicator of curd maturity (Chinese standard for locally marked products of sufu marked products of sufu (2015) (DB53/T 713-2015)). The content of amino acid nitrogen in natural and inoculated sufu gradually increased with the extension of fermentation time Figure 2F, which is consistent with the results of Cai [23]. At 90 days of post-fermentation, the amino acid nitrogen content in natural and inoculated sufu reached 0.66 g/100 g and 0.55 g/100 g, respectively, both of which reached the standard for maturation of sufu. After 180 days of post-fermentation, the amino acid nitrogen reached 0.80 g/100 g and 0.74 g/100 g, respectively.

### 3.2. Protein Hydrolysis

The protein in sufu could be degraded into small molecular peptides, free amino acids and ammonia gas during fermentation, which had an important role in the formation of the texture and flavor of sufu. The degree of protein hydrolysis in sufu was characterized using the water-soluble protein content/total protein content (WP/TP) and amino acid nitrogen content/total nitrogen content (AAN/TN). As shown in Table 2, the degree of protein hydrolysis of both types of sufu gradually increased with increased fermentation time. At the same fermentation time, the degree of hydrolysis of protein in naturally fermented sufu was slightly higher than that of inoculated fermented sufu, indicating the greater ability of microorganisms in naturally fermented sufu to secrete proteases that hydrolyze the protein.

### 3.3. Free Amino Acids

Free amino acids (FAA) are an important taste source in fermented food. They mainly contribute to umami, sweetness and bitterness: Glutamic acid and Aspartic acid contributes to umami; Proline, Alanine, Serine, Lysine, Glycine, Valine and Threonine promote sweetness; while Leucine, Phenylalanine, Tyrosine, Isoleucine, Histidine, Arginase and Methionine contribute to bitterness [15,24,25,26]. In addition, they are also an important precursor for the formation of volatile flavor components [27]. The changes of FAA in sufu fermented using both natural and inoculated bacteria are shown in Table 3. The FAA in both types of sufu increased with the prolongation of fermentation time. Glu, an umami-flavored FAA, was apparently the most abundant of all these FAA, which was consistent with the previous results [24]. Before fermentation (0 day), the total FAA in two types of tofu were 5.16 and 5.04 mg/g, respectively, and this amount was around 5 and 7.5-fold higher after 90 and 180 days of fermentation, respectively. In the post-fermentation stage, there was no significant difference (*p* < 0.05) in the total amount of FAA in both natural and inoculated fermented sufu.

These FAA affected the taste profile when the ratio of concentration and threshold (TAV values) of these compounds was >1. In addition, the taste compounds will also affect the flavor through the synergistic effects of these compounds [28]. Table 4 indicates the FAA TAV values in both natural and inoculated fermented sufu. There were three amino acids with TAV values > 1 in natural and inoculated fermented sufu before fermentation, namely, Glu, Ala and His, which indicated their greater contribution to the overall taste profile. The TAV values of the other 13 amino acids were all <1, indicating that their contribution to the taste was not prominent. After 90 days post-fermentation, the number of TAV values >1 increased to 10 in both natural and inoculated fermented sufu, with the remaining 6 amino acids <1. After 180 days post-fermentation, the number of FAA with TAV values > 1 was higher in naturally fermented sufu than inoculated fermented sufu. Among all these FFA, Glu with an umami taste had the highest TAV value, followed by Val and Lys with sweet tastes. The synergetic function of these flavor amino acids mostly accounts for the unique flavor of sufu. In general, with the prolongation of fermentation, the content of FAA in sufu increased significantly, resulting in a stronger taste. There was no significant difference (*p* < 0.05) in the total amount of FAA between natural and inoculated fermented sufu at the same fermentation stage.

### 3.4. Textual and Structural Properties

TPA analysis was carried out on both natural and inoculated sufu, and the results are shown in Table 5. The hardness of both types of sufu decreased during the fermentation, with inoculated fermented sufu higher than naturally fermented sufu, which was consistent with Xia, Li, Zheng, Ran and Kan [29]. This reduction may result from the enzymatic hydrolysis of the three-dimensional protein network [30], which also led to increased FAA, as shown in Table 2. The texture of sufu could be an indicator of sufu ripening [31,32]. A similar trend was observed for the changes in adhesiveness, which was consistent with Zhao and Zheng [33]. However, the type and time of fermentation had no significant effect on the springiness of sufu.

**Figure 3:**
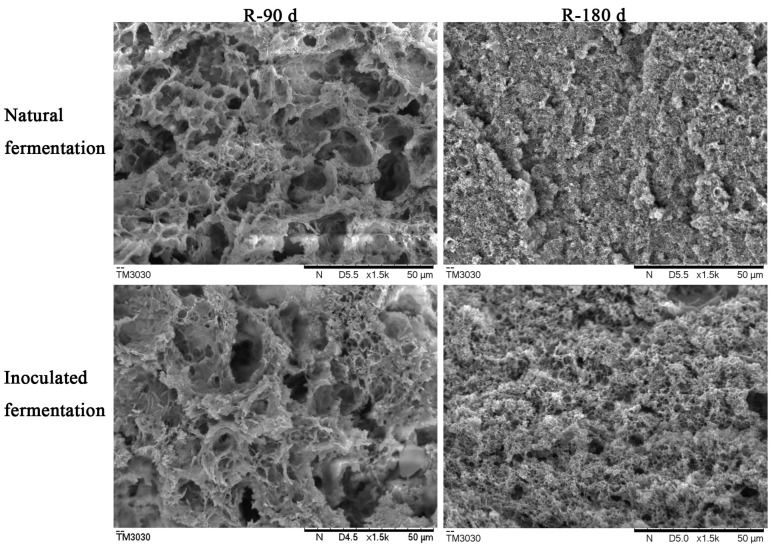
SEM micrographs of natural and inoculated fermented sufu after 90 and 180 d of fermentation.

The microstructure of natural and inoculated fermented sufu at 90 and 180 days post-fermentation was observed using SEM (Figure 3). With the increase of fermentation time, the internal diameter of both types of sufu gradually decreased, and the internal structure became smooth, which was consistent with previous reports [33,34]. This may be related to protein hydrolysis. After 90 days post-fermentation, the interior of sufu had a network structure with more large pores, which may be due to the low degree of protein hydrolysis. The colloidal particles of protein were large and the damage to the protein gel structure was limited. After 180 days post-fermentation, both types of sufu had a significantly smaller pore size and were denser and more uniform than at 90 days, which may be related to the greater degree of hydrolysis of protein. At the same fermentation time, the internal gel pore size of naturally fermented sufu was slightly smaller than that of inoculated fermented sufu, indicating that the degree of protein hydrolysis in naturally fermented sufu was slightly higher, which was consistent with data in Table 1.

### 3.5. Aroma Profiles

**Figure 4:**
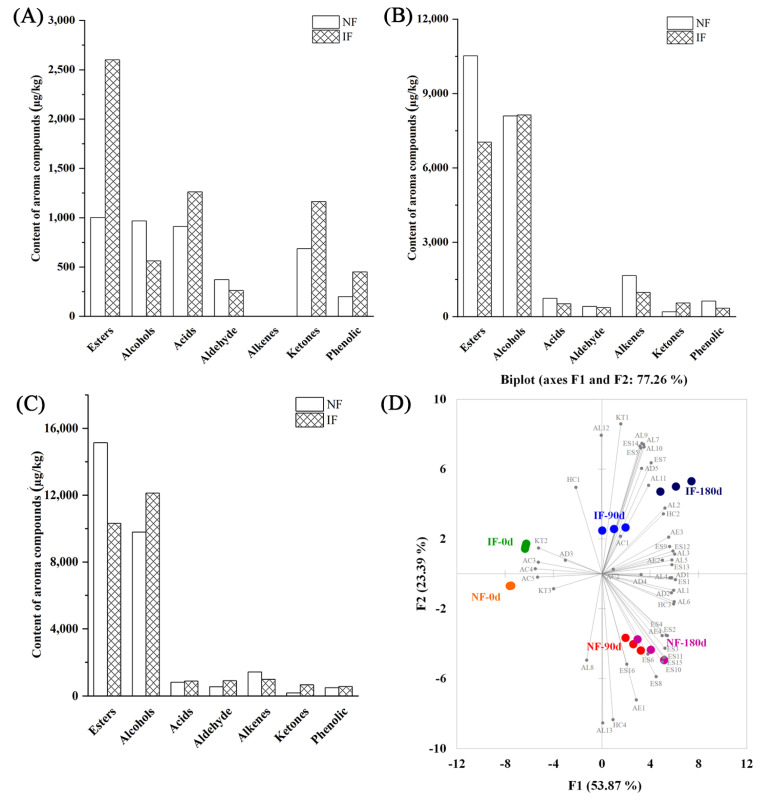
Comparison of aroma compounds between natural and inoculated fermented sufu at 0 d (**A**), 90 d (**B**) and 180 d (**C**) of fermentation and PCA analysis (**D**). NF: natural fermentation; IF: inoculated fermentation. Note: ES1: ethyl octanoate; ES2: ethyl palmitate; ES3: ethyl linoleate; ES4: ethyl acetate; ES5: 5−2−phenylethyl propionate; ES6: 14,17−octadecyl methyl dienoate; ES7: ethyl lactate; ES8: methyl 9,12,15−octadecatrienoate; ES9: ethyl benzoate; ES10: ethyl myristate; ES11: linalyl acetate; ES12: gamma nonalactone; ES13: ethyl phenylacetate; ES14: isoamyl acetate; ES15: methyl salicylate; ES16: ethyl elaidate; AL1: linalool; AL2: ethanol; AL3: isoamyl alcohol; AL4−: terpene alcohol; AL5: 1-pentanol; AL6: terpineol; AL7:2−nonylalcohol;AL8:trans−3−(4−hydroxy−3−methoxylphenyl)−2−propen−1−ol;AL9:cis−A,α−5−trimethyl−5−vinyltetrahydrofuran−2−methanol; AL10: nerol; AL11: 2,4−decadien−1−ol; AL12: benzyl alcohol; AL13: phenethyl alcohol; AC1: stearic acid; AC2: palmitic acid; AC3: octanoic acid; AC4: caproic acid; AC5: 4−methylvalericacid;AD1:benzene formaldehyde;AD2: (9ci)−2,6,6−trimethyl−1,4−cyclohexadiene−1−carboxaldehyde; AD3: phenylacetaldehyde; AD4: eicosanal; AD5: 2−hexane alkenal; AE1: limonene; AE2: ocimene; AE3: myrcene; AE4: cypressene; KT1: thujone; KT2: 2−nonanone; KT3: 3−octanone; HCl: methylcyclopentane; HC2: 2−methoxy−4−methylphenol; HC3: 2,3−dihydrobenzofuran; and HC4: indole.

The aroma compounds in natural and inoculated sufu at 0, 90 and 180 d post-fermentation were obtained using GC-MS (Table 6 and Figure 4). Esters, alcohols, acids, aldehydes and alkenes were the main aroma components in the fermented sufu. The total number of aroma compounds increased continuously for both types of sufu with fermentation, while the total number of aroma compounds increased significantly after 90 days and did not change at 180 days. Esters and alcohols were among the most numerous aroma compounds, which was consistent with previous studies [1,12]. At the beginning of fermentation, added alcohol could react with acids, such as free fatty acids, through esterification, which resulted in the increase of both the amount and number of esters. After a period of fermentation, the number of esters had been maximized but the amount kept increasing. The content of esters was positively correlated with the added alcohol content [7]. Most of the esters have a unique aroma, which can give the sufu a sweet smell. Among them, ethyl caprylate, ethyl palmitate, ethyl linoleate, ethyl acetate, 2-phenylethyl propionate and ethyl phenylacetate are associated with fruity, milky and floral flavors. Ethyl benzoate and methyl salicylate contributed wintergreen oil aroma, and ethyl myristate and gamma nonalactone contributed a coconut aroma [35]. With the increase of fermentation time, the content of aroma compounds in natural sufu was higher than inoculated sufu, while the number of aroma compounds was lower.

Principal component (PC) analysis of aroma compounds of natural and inoculated fermented sufu at 0, 90 and 180 days post-fermentation is shown in Figure 3D. The sufu with different fermentation strains and fermentation times could be distinguished and the contribution percentages of PC 1 and 2 were 53.9 and 23.4%, respectively. Among them, PC 1 was mainly positively correlated with ES1-ES15, AL1-AL7, AL9, AL10, AL12, AC3, AC4, AC5, AD1, AD2, AD5, AE2-AE4, KT2 and HC1-HC3, while PC 2 was positively correlated with AL8, AL11, AC2, AD3, AD4 and AE1. Sufu fermented with different strains could be distinguished by PC 1, while the sufu with different fermentation times with the same strains were significantly differentiated by PC 2.

### 3.6. Microbiology

**Figure 5:**
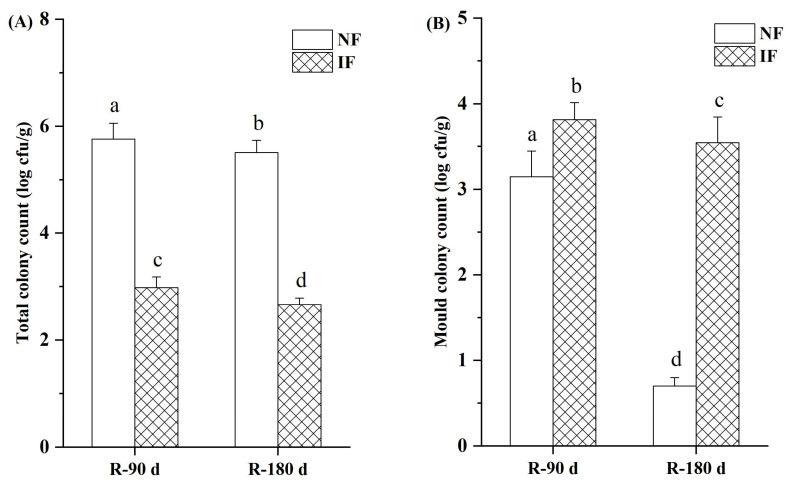
The total colony count (**A**) and mold colony count (**B**) in natural and inoculated fermented sufu at 90 and 180 d of fermentation. Note: Different lower-case letters indicated significant differences at *p* < 0.05. NF: natural fermentation; IF: inoculated fermentation.

Figure 5 shows the changes of the total number of colonies and the content of mold colonies during natural and inoculated sufu fermentation at 90 and 180 days. The total number of colonies and mold colonies in natural sufu decreased significantly with increased fermentation time, which was consistent with Han [4]. The total number of colonies in natural sufu was significantly higher than that in inoculated sufu, while the number of mold colonies was always lower than that in inoculated sufu. The content of pathogenic bacteria, such as *Salmonella*, *S. aureus* and *E. coli*, in natural and inoculated sufu was not detectable (data not shown), which meant that the samples were free of these pathogens.

### 3.7. Biogenic Amines Content

**Figure A1:**
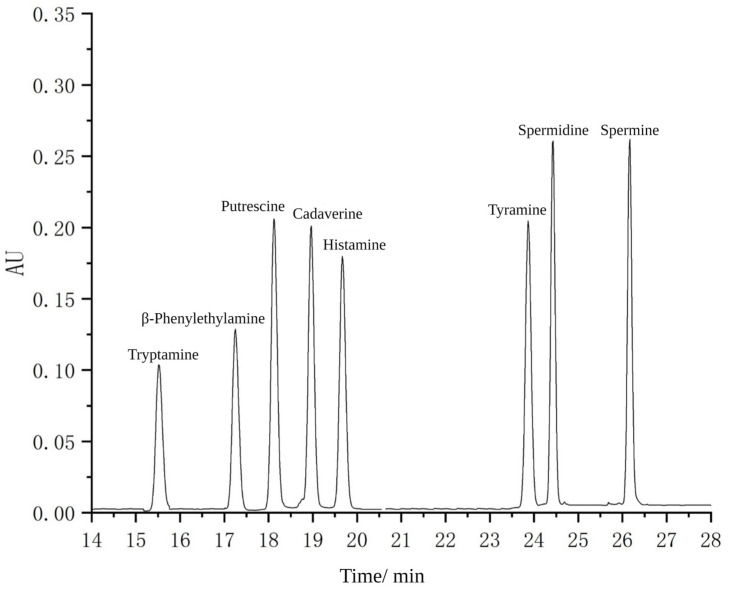
Chromatogram of derivatives of biogenic amines standards (50 µg/mL).

The HPLC chromatograms of the biogenic amine standard derivatives and the regression equation tables for the linearity of each biogenic amine derivative are shown in Figure A1 and Table A1. The biogenic amine contents of natural and inoculated sufu at post-fermentation 0 days, 90 days and 180 days are shown in Table 7. The total biogenic amine content in sufu tended to increase gradually with the increase of fermentation time. The main biogenic amines in sufu were putrescine, cadaverine, histamine and spermidine, among which, putrescine and cadaverine were associated with unhygienic environmental conditions of food processing [36]. Thus, it can be seen that the hygienic conditions of sufu production and processing still need to be improved. The contents of β-phenylethylamine, putrescine, tyramine and total biogenic amines in inoculated sufu were significantly lower than those in natural sufu (*p* < 0.05), probably because of the stronger activity of amino acid decarboxylase produced by microorganisms in inoculated sufu. Notably, the histamine content in inoculated sufu was significantly higher than that in natural sufu (*p* < 0.05), but all were lower than 100 mg/kg, and Nout [37] concluded that the histamine content in foods within the range of 50–100 mg/kg could be considered as the production process meeting the requirements of good practice. The production of histamine can be effectively inhibited by inhibiting the activity of histidine decarboxylase in foods, and Kang [38,39] found that citric acid, malic acid, sorbic acid, succinic acid and sorbitol significantly inhibited the activity of histidine decarboxylase and suppressed the production of histamine in mackerel.

## 4. Conclusions

After 90 days of fermentation, both naturally fermented and inoculated fermented sufu reached the maturity standard of sufu. Compared with the naturally fermented sufu, the inoculated sufu had less protein hydrolysis, higher hardness, larger pore sizes and a lower number of aroma compounds, while there was no significant difference for FAA, and both were free of the tested pathogens. Inoculated fermented sufu had a lower content of total biogenic amines than naturally fermented sufu. Especially in inhibiting histamine production, inoculated fermentation has a better effect in decreasing histamine content compared with natural fermentation. The quality of sufu might be further optimized by inoculating using mixed strains since this study only used *Mucor racemosa M2*.

In summary, the M2 strain was used to prepare inoculated fermented sufu, and the differences in quality between naturally fermented sufu and inoculated fermented sufu at 90 and 180 d were investigated. The quality of inoculated sufu was somewhat better than that of natural sufu. Finally, we determined the feasibility of the M2 strain as a sufu fermentation strain.

## Figures and Tables

**Table 1:** Gradient elution program.

Time/Min	Mobile Phase A(%)	Mobile Phase B(%)
0	60	40
2	60	40
5	38	62
10	38	62
16	20	80
20	20	80
21	0	100
25	0	100
27	60	40
40	60	40

**Table 2:** The degree of hydrolysis of protein in natural and inoculated fermented sufu.

**Sample**	**Natural Fermentation**			**Inoculated Fermentation**		
	R-0 d	R-90 d	R-180 d	R-0 d	R-90 d	R-180 d
WP/TP (%)	17.3 ± 0.1 ^c^	34 ± 1 ^b^	40 ± 1 ^a^	16.6 ± 0.2 ^c^	28.2 ± 0.4 ^b^	40 ± 1 ^a^
AAN/T (%)	13.0 ± 0.4 ^c^	33 ± 1 ^b^	39 ± 1 ^a^	15.4 ± 0.2 ^c^	27 ± 1 ^b^	36 ± 1 ^a^

Note: Means in a row followed by different lower-case letters differ significantly (*p* < 0.05). WP/TP: water-soluble protein/total protein; AAN/TN: amino acid nitrogen/total nitrogen.

**Table 3:** Contents of FAA in natural and inoculated fermented sufu.

FAA	Taste	Contents of FAA (mg/g)					
		R-0 d		R-90 d		R-180 d	
		Natural Fermentation	Inoculated Fermentation	Natural Fermentation	Inoculated Fermentation	Natural Fermentation	Inoculated Fermentation
Asx	Umami	0.40 ± 0.01 ^c^	0.44 ± 0.01 ^c^	2.3 ± 0.3 ^b^	2.36 ± 0.04 ^b^	4.3 ± 0.2 ^a^	4.3 ± 0.6 ^a^
Glx	Umami	1.00 ± 0.02 ^a^	1.07 ± 0.01 ^a^	5.9 ± 0.6 ^c^	4.8 ± 0.1 ^d^	9.2 ± 0.4 ^a^	7.6 ± 1.0 ^b^
Ser	Sweet	0.07 ± 0.00 ^d^	0.11 ± 0.00 ^d^	0.60 ± 0.01 ^bc^	0.67 ± 0.19 ^b^	0.52 ± 0.03 ^c^	1.26 ± 0.03 ^a^
Gly	Sweet	0.15 ± 0.00 ^d^	0.11 ± 0.01 ^d^	1.0 ± 0.1 ^c^	1.1 ± 0.5 ^bc^	1.8 ± 0.1 ^a^	1.4 ± 0.2 ^b^
Thr	Sweet	0.20 ± 0.00 ^c^	0.18 ± 0.01 ^c^	1.30 ± 0.06 ^b^	1.29 ± 0.05 ^b^	1.94 ± 0.08 ^a^	1.85 ± 0.18 ^a^
Ala	Sweet	0.65 ± 0.01 ^e^	0.63 ± 0.01 ^e^	1.67 ± 0.17 ^c^	1.24 ± 0.08 ^d^	2.50 ± 0.09 ^a^	1.97 ± 0.26 ^b^
Lys	Sweet	0.44 ± 0.01 ^e^	0.34 ± 0.01 ^e^	1.95 ± 0.21 ^c^	1.67 ± 0.17 ^d^	3.24 ± 0.19 ^a^	2.84 ± 0.38 ^b^
Val	Sweet	0.29 ± 0.00 ^e^	0.30 ± 0.01 ^e^	1.63 ± 0.17 ^c^	1.38 ± 0.04 ^d^	2.64 ± 0.13 ^a^	2.35 ± 0.27 ^b^
Pro	Sweet	0.25 ± 0.01 ^d^	0.28 ± 0.03 ^d^	1.62 ± 0.16 ^b^	1.46 ± 0.11 ^b^	0.74 ± 0.09 ^c^	2.34 ± 0.35 ^a^
Phe	Bitter	0.38 ± 0.01 ^c^	0.32 ± 0.00 ^c^	1.71 ± 0.19 ^b^	1.56 ± 0.12 ^b^	2.66 ± 0.14 ^a^	2.44 ± 0.35 ^a^
Leu	Bitter	0.37 ± 0.01 ^d^	0.33 ± 0.01 ^d^	2.24 ± 0.24 ^c^	2.02 ± 0.07 ^c^	3.72 ± 0.16 ^a^	3.34 ± 0.46 ^b^
Tyr	Bitter	0.23 ± 0.00 ^c^	0.20 ± 0.00 ^c^	0.71 ± 0.01 ^b^	0.66 ± 0.06 ^b^	0.90 ± 0.13 ^a^	0.85 ± 0.19 ^a^
Arg	Bitter	0.21 ± 0.00 ^a^	0.17 ± 0.01 ^a^	0.10 ± 0.01 ^b^	0.21 ± 0.08 ^a^	0.03 ± 0.03 ^c^	0.04 ± 0.00 ^c^
Ile	Bitter	0.23 ± 0.01 ^e^	0.22 ± 0.02 ^e^	1.54 ± 0.16 ^c^	1.30 ± 0.03 ^d^	2.55 ± 0.11 ^a^	2.26 ± 0.31 ^b^
His	Bitter	0.21 ± 0.00 ^e^	0.23 ± 0.02 ^e^	0.64 ± 0.06 ^c^	0.48 ± 0.05 ^d^	0.94 ± 0.01 ^a^	0.84 ± 0.10 ^b^
Met	Bitter	0.06 ± 0.00 ^c^	0.08 ± 0.00 ^c^	0.39 ± 0.05 ^b^	0.34 ± 0.00 ^b^	0.63 ± 0.05 ^a^	0.58 ± 0.08 ^a^
Total		5.2 ± 0.1 ^c^	5.04 ± 0.00 ^c^	25 ± 2 ^b^	22 ± 1 ^b^	38 ± 2 ^a^	36 ± 5 ^a^

Note: Means in a row followed by different lower-case letters differ significantly (*p* < 0.05). Asx means Asp and Asn; Glx means Glu and Gln. Ser: Serine; Gly: Glycine; Thr: Threonine; Ala: Alanine; Lys: Lysine; Val: Valine; Pro: Proline; Phe: Phenylalanine; Leu: Leucine; Tyr: Tyrosin;, Arg: Arginase; Ile: Isoleucine; His: Histidine; Met: Methionine.

**Table 4:** TAV of free amino acids in natural and inoculated fermented sufu.

FAA	Taste	Taste Threshold [27] (mg/g)	R-0 d		R-90 d		R-180 d	
			Natural Fermentation	Inoculated Fermentation	Natural Fermentation	Natural Fermentation	Inoculated Fermentation	Natural Fermentation
Asx	Umami	1.00	0.40	0.44	2.31	2.36	4.34	4.28
Glx	Umami	0.30	3.35	3.58	19.59	16.01	30.76	25.18
Ser	Sweet	1.50	0.05	0.07	0.40	0.44	0.35	0.84
Gly	Sweet	1.30	0.12	0.09	0.79	0.86	1.39	1.06
Thr	Sweet	2.60	0.08	0.07	0.50	0.50	0.75	0.71
Ala	Sweet	0.60	1.08	1.06	2.79	2.07	4.17	3.28
Lys	Sweet	0.50	0.88	0.69	3.90	3.33	6.48	5.68
Val	Sweet	0.40	0.73	0.76	4.07	3.45	6.61	5.88
Pro	Sweet	3.00	0.08	0.09	0.54	0.49	0.25	0.78
Phe	Bitter	0.90	0.42	0.36	1.90	1.73	2.96	2.71
Leu	Bitter	1.90	0.20	0.17	1.18	1.07	1.96	1.76
Tyr	Bitter	0.90	0.26	0.22	0.79	0.73	1.00	0.94
Arg	Bitter	0.50	0.43	0.34	0.21	0.43	0.06	0.09
Ile	Bitter	0.90	0.26	0.24	1.71	1.45	2.83	2.51
His	Bitter	0.20	1.06	1.16	3.18	2.40	4.71	4.18
Met	Bitter	0.30	0.20	0.28	1.31	1.14	2.10	1.92

Note: Asx means Asp and Asn; Glx means Glu and Gln. Ser: Serine; Gly: Glycine; Thr: Threonine; Ala: Alanine; Lys: Lysine; Val: Valine; Pro: Proline; Phe: Phenylalanine; Leu: Leucine; Tyr: Tyrosine; Arg: Arginase; Ile: Isoleucine; His: Histidine; Met: Methionine.

**Table 5:** Texture profile analysis of natural and inoculated fermented sufu.

Time	Sample	Hardness/g	Adhesiveness/g	Springiness/%
R-90 d	Natural fermentation	790 ± 57 ^b^	−23 ± 28 ^b^	0.52 ± 0.08 ^a^
	Inoculated fermentation	1063 ± 211 ^a^	−80 ± 47 ^a^	0.60 ± 0.06 ^a^
R-180 d	Natural fermentation	508 ± 45 ^c^	−56 ± 44 ^ab^	0.53 ± 0.10 ^a^
	Inoculated fermentation	697 ± 58 ^b^	−100 ± 82 ^a^	0.52 ± 0.09 ^a^

Note: Means in a column followed by different lower-case letters differ significantly (*p* < 0.05).

**Table 6:** Comparison of aroma compound types from natural and inoculated fermented sufu.

Time	Sample	Esters	Alcohols	Acids	Aldehyde	Alkenes	Ketones	Phenolics	Total
R-0 d	Natural fermentation	3	4	5	3	0	2	3	20
	Inoculated fermentation	5	6	5	4	0	2	3	25
R-90 d	Natural fermentation	13	9	5	4	4	1	3	39
	Inoculated fermentation	15	12	5	4	4	2	3	45
R-180 d	Natural fermentation	14	9	5	4	4	1	3	40
	Inoculated fermentation	15	12	5	5	3	2	3	45

**Table 7:** Biogenic amine content in natural and inoculated fermented sufu at 0 d, 90 d and 180 d of fermentation.

Biogenic Amines	R-0 d		R-90 d		R-180 d	
	Natural Fermentation	Inoculated Fermentation	Natural Fermentation	Inoculated Fermentation	Natural Fermentation	Inoculated Fermentation
Tryptamine	7.81 ± 0.42 ^d^	14.84 ± 0.58 ^a^	3.40 ± 0.00 ^e^	12.37 ± 0.92 ^b^	9.22 ± 0.24 ^c^	12.03 ± 0.23 ^b^
β-Phenylethylamine	-	1.97 ± 0.92 ^c^	16.61 ± 1.50 ^b^	2.24 ± 0.80 ^c^	21.19 ± 2.69 ^a^	2.19 ± 0.06 ^c^
Putrescine	138.24 ± 1.69 ^c^	75.55 ± 0.53 ^d^	187.42 ± 6.90 ^a^	80.82 ± 6.13 ^d^	166.51 ± 15.33 ^b^	84.77 ± 0.67 ^d^
Cadaverine	30.13 ± 1.69 ^b^	24.30 ± 4.31 ^b^	109.33 ± 13.54 ^a^	110.28 ± 4.29 ^a^	103.68 ± 6.18 ^a^	107.04 ± 3.57 ^a^
Histamine	18.70 ± 1.72 ^e^	29.14 ± 2.09 ^d^	44.24 ± 0.71 ^c^	64.95 ± 4.55 ^b^	66.15 ± 3.33 ^b^	85.99 ± 0.78 ^a^
Tyramine	6.47 ± 0.30 ^c^	2.50 ± 0.76 ^c^	163.57 ± 16.78 ^b^	2.28 ± 0.58 ^c^	196.29 ± 14.51 ^a^	4.97 ± 0.10 ^c^
Spermidine	1.84 ± 0.48 ^e^	-	7.88 ± 0.60 ^c^	5.07 ± 0.74 ^d^	14.82 ± 1.21 ^a^	10.91 ± 0.83 ^b^
Spermine	90.29 ± 3.19 ^c^	97.05 ± 3.67 ^b^	79.94 ± 6.87 ^d^	73.98 ± 0.03 ^d^	100.60 ± 8.78 ^ab^	105.71 ± 0.78 ^a^

Note: Means in a column followed by different lower-case letters differ significantly (*p* < 0.05).

## Data Availability

Data are contained within the article.

## Appendix A

**Table A1:** Regression equations of biogenic amines derivatives.

Biogenic Amines	Regression Equation	R^2^	Linearity Range (µg/mL)
Tryptamine	y = 22,539x − 6175	0.9999	0.1–80
β-Phenylethylamine	y = 26,594x + 2680.7	0.9998	0.1–80
Putrescine	y = 41,619x − 13,198	0.9995	0.1–80
Cadaverine	y = 38,533x + 10,414	0.9998	0.1–80
Histamine	y = 35,779x + 5025.7	0.9998	0.1–80
Tyramine	y = 39,721x + 24,141	0.9994	0.1–80
Spermidine	y = 38,922x + 73,484	0.9999	0.1–80
Spermine	y = 36,165x + 86,728	1.0000	0.1–80
